# A phase II study to evaluate the efficacy of commercially available molecularly matched targeted therapies in the second-line setting

**DOI:** 10.1093/oncolo/oyag279

**Published:** 2026-07-22

**Authors:** Suzanne Jones, Gerald Falchook, Stephanie Graff, Edward Arrowsmith, Deepak Kilari, Todd A Gersten, Maen Hussein, Ivor Percent, Howard A Burris

**Affiliations:** Sarah Cannon Research Institute, Nashville, TN 37203, United States; Sarah Cannon Research Institute at HealthOne, Denver, CO 80218, United States; HCA Midwest Kansas City, Kansas City, KS 64132, United States; Tennessee Oncology, PLLC, Nashville, TN 37203, United States; Medical College of Wisconsin, Milwaukee, WI 53226, United States; Florida Cancer Specialists and Research Institute East, Wellington, FL 33414, United States; Sarah Cannon Research Institute at Florida Cancer Specialists and Research Institute North, Tavares, FL 32778, United States; Sarah Cannon Research Institute at Florida Cancer Specialists and Research Institute South, North Port, FL 34286, United States; Sarah Cannon Research Institute, Nashville, TN 37203, United States; SCRI Oncology Partners, Nashville, TN 37203, United States

**Keywords:** targeted therapy, precision oncology, regorafenib, afatinib, cabozantinib

## Abstract

**Background:**

Initial studies have shown improved outcomes in patients receiving cancer therapies matched to their molecular alterations. To improve the chances of finding a therapeutic match for patients, this study evaluated the preliminary antitumor activity of 3 commercially available multitargeted agents in the United States, regorafenib, afatinib, and cabozantinib.

**Methods:**

In this phase II trial, patients who did not benefit from first-line treatment for non-small cell lung cancer (NSCLC), upper aerodigestive tract cancers, non-colon gastrointestinal cancers, and urothelial carcinoma underwent next-generation sequencing to identify actionable genomic alterations. Eligible patients, based on identified mutations deemed treatable by regorafenib, cabozantinib, or afatinib, were enrolled to receive matched targeted therapies. Outcomes were monitored via Response Evaluation Criteria in Solid Tumors criteria, with dose modifications per National Cancer Institute Common Terminology Criteria for Adverse Events, v4.03 guidelines for adverse events (AEs).

**Results:**

One hundred patients with metastatic cancers were enrolled across tumor types. Median treatment durations were 12 weeks (regorafenib), 10.7 weeks (afatinib), and 24.1 weeks (cabozantinib). The overall objective response rate was 7.0%, with 1 complete response and 8 partial responses. The clinical benefit rate, including responses and stable disease >6 months, was 16.0%. Median progression-free survival ranged from 1.9 months for urothelial carcinoma to 3.2 months for NSCLC. Toxicities were common for all medications; for regorafenib, 90.7% of patients had AEs (50% Grade 3/4); for afatinib, 86% of patients had AEs (54% Grade 3/4); for cabozantinib, 100% of patients had AEs (36% Grade 3/4). The most common AEs were diarrhea, fatigue, nausea, decreased appetite, and stomatitis.

**Conclusions:**

Regorafenib, afatinib, and cabozantinib had modest effects when used as molecularly matched targeted therapies in patients with NSCLC, upper aerodigestive tract cancers, non-colon gastrointestinal cancers, and urothelial carcinoma in the second-line setting. Future research could examine more precise matching of therapies to genomic alterations and evaluate combination therapies.

Implications for Practice Regorafenib, afatinib, and cabozantinib had modest effects when used as molecularly matched targeted therapies in patients with non-small cell lung cancer, upper aerodigestive tract cancers, non-colon gastrointestinal cancers, and urothelial carcinoma in the second-line setting. To advance the evidence base for precision oncology, future research could examine approaches that more precisely match available therapies to genomic alterations and that combine targeted therapies or include immunotherapy.

## Introduction

Precision medicine in cancer treatment uses genomic profiling to identify mutations causing cancer and matches them to targeted drug therapies. This approach has shown promising results in treating certain cancers such as lung carcinoma and melanoma.^1–3^ For *EGFR* mutant non-small cell lung cancer (NSCLC)^4^ and *BRAF* mutant melanoma,^5^ targeted drug therapies have become standard treatments. However, the use of genomic profiling to identify patients showing an excellent response to treatment in rare cancers remains unclear, and the broad-scale use of genomic profiling covering a wider range of cancers needs further systematic research.^6^

Precision medicine studies that focus mainly on patients showing an excellent response treatment may underscore the benefits seen when this approach is used in larger patient populations. In a pilot study by Von Hoff et al,^7^ patients from 9 different centers were molecularly profiled; a molecular target was detected in 98% of patients, and 79% of these patients were treated with the regimen recommended by the molecular profiling. The targeted regimen resulted in longer progression-free survival (PFS) (relative to the most recent therapy on which the patient experienced progression) in 27% of patients.^7^ In another analysis, which used the Foundation One test, Johnson et al^8^ found that approximately 83% of patients with advanced stages of cancer had potentially actionable genetic alterations; 21% of patients received targeted therapy. Furthermore, 26% of patients had genetic alterations that were responsive to therapies that were already approved and were commercially available.^8^

Promisingly, prices for genomic testing continue to decline,^6^ and insurance coverage for molecular testing has expanded.^9^ However, the use of targeted therapies is still limited by the challenges of information processing, genomic medical education and interpretation, cost, and reimbursement.^10^ Further large-scale studies examining a range of cancer types are needed to refine the concept of using molecular profiling and targeted therapies. Building on previous studies that have established the feasibility of conducting such research,^11–14^ we undertook a multicenter, open-label phase II clinical trial using 3 commercially available, multitargeted, multicancer drugs, regorafenib, afatinib, and cabozantinib, matched to patients who did not benefit from first-line treatment for NSCLC (patients with *ALK* or *EGFR* mutations were ineligible due to the availability of approved targeted agents for use in the second-line setting), upper aerodigestive tract cancers, non-colon gastrointestinal cancers, and urothelial carcinoma. These 3 multitargeted tyrosine kinase agents were chosen as therapeutic options to increase the likelihood of matching to patients with relapsed or refractory cancer due to their known activity against a variety of mutations or molecular aberrations, thereby enabling the recruitment of a sufficient study population.

## Patients and method

### Study design

This 4-arm pilot Phase II study, which began on October 5, 2016 and concluded on August 15, 2022, evaluated the preliminary antitumor activity of regorafenib, afatinib, and cabozantinib in patients who had disease progression after first-line treatment for 1 of the following tumor types: NSCLC, upper aerodigestive tract cancers, non-colon gastrointestinal cancers, and urothelial carcinoma (NCT02795156). Patients underwent genetic tests using next-generation sequencing (NGS) for their tumors to identify certain genomic alterations that could be targeted by regorafenib, afatinib, or cabozantinib, which were already approved by the US Food and Drug Administration for other medical conditions. The medications were administered according to standard approved dosage guidelines. Patients were reevaluated for treatment response after receiving 8 weeks of oral therapy (except for regorafenib, which was dosed 21 days on followed by 7 days off for a 28-day cycle; afatinib and cabozantinib were both dosed daily) and continued treatment with reevaluations every 8 weeks until the time of progressive disease or intolerable treatment-related adverse events (TRAEs).

### Eligibility

The study included patients aged ≥18 years with a histologically or cytologically confirmed diagnosis of 1 of the following tumor types whose disease had progressed following 1 line of standard therapy and/or for which no standard treatment was available that had been shown to prolong survival: NSCLC, upper aerodigestive tract cancers (including lip, tongue, salivary gland, gum, oral cavity, mouth, tonsils, oropharynx, nasopharynx, nasal cavity, sinus, and larynx tumors), non-colon gastrointestinal cancers (including hepatobiliary, pancreatic, and gastroesophageal tumors), and urothelial carcinoma. If a commercially available and approved therapy was available for the mutation or alteration being targeted, the patient was not eligible for participation in the study. Patients were required to have 1 of the genetic mutations needed for the study. Patients were allowed to have received only 1 prior chemotherapy regimen in the metastatic setting. Patients were allowed to receive unlimited prior non-chemotherapy therapies. Patients were required to have an Eastern Cooperative Oncology Group (ECOG) Performance Status score of 0 or 1.

Adequate hematologic function was defined as an absolute neutrophil count ≥1500/μL and platelets ≥75,000/μL. Adequate liver function was defined as alanine aminotransferase and aspartate aminotransferase ≤2.5 × the upper limit of normal (ULN) or ≤5 × ULN if liver metastases were present, and total bilirubin ≤1.5 × ULN (unless the patient had Grade 1 bilirubin elevation due to Gilbert’s disease or a similar syndrome involving slow conjugation of bilirubin). Adequate renal function was defined as serum creatinine ≤1.5 × ULN or measured or calculated creatinine clearance ≥50 mL/min for patients with creatinine levels ≥1.5 × ULN. Patients who were treated with an agent such as warfarin or heparin were allowed to receive either regorafenib or afatinib, provided that their medication dose and international normalized ratio/partial thromboplastin time were stable, with close monitoring mandatory if the patient was receiving anticoagulants. Male patients with female partners of childbearing potential and female patients of childbearing potential were required to use 2 forms of acceptable contraception, including 1 barrier method, during their participation in the study and for 90 days following the last dose of study drug; male patients were also required to refrain from donating sperm during this period. Additionally, willingness and ability to comply with study and follow-up procedures, and the ability to understand the nature of this study and give written informed consent were necessary.

### Pretreatment evaluation

To identify patients with tumors likely to respond to the study treatment, pre-enrollment tumor sequencing was conducted using tissue or blood samples. Patients underwent NGS testing as part of standard-of-care management outside of the trial; NGS testing from either time of initial diagnosis or in the relapsed setting was permissible for inclusion in the study. Foundation One testing was only performed in any patient whose standard-of-care testing was not done via Foundation Medicine (if there was still tissue available to be tested). Patients were enrolled based on the report from their standard-of-care testing. Patients with NSCLC who had *ALK* or *EGFR* mutations were excluded since standard therapies already exist. Patients with *RAS* or *BRAF* mutations received appropriate matched inhibitors when suitable. Before starting therapy, patients underwent a complete medical evaluation, including medical history, physical examination, bloodwork, cardiac evaluation, and pregnancy screening as needed. Computed tomography imaging of the chest, abdomen, pelvis, and brain (if symptomatic) was done to visualize tumors and establish baseline measurements.

### Treatment regimen

Patients received regorafenib, afatinib, or cabozantinib, matching their tumor genetic mutation. As these agents were used off-label, they were provided free of cost by the drug manufacturers. Patients took their assigned oral study drug daily at the recommended dose, except for regorafenib, which followed a 21-day-on/7-day-off schedule (Table 1). Missed doses or vomiting were managed by resuming the regular regimen without replacement of the missed/vomited dose. Antiemetic medication was used only as needed. Adherence was monitored by pill counts on day 1 of each 28-day cycle. Once the dose was reduced due to a TRAE for a patient, the dose remained decreased for the duration of therapy. Patients requiring a dosage interruption of >28 days were removed from the study. Response assessment with imaging and/or labs occurred every 8 weeks. Treatment continued until progressive disease or investigator-recommended discontinuation. Patients could discuss any additional medications with the study team.

**Table 1.** FDA-approved targeted agents.

Approved drug	Targets listed in package insert	Manufacturer	Recommended dose
**Regorafenib (Stivarga)**	*RET, VEGF1-3, KIT, PDGFR-α and β, FGFR1-2, TIE2, DDR2, Trk2A, Eph2A, RAF-1, BRAF, SAPK2, PTK5, Abl*	Bayer	160 mg once daily for the first 21 days of each 28-day cycle
**Afatinib (Gilotrif)**	*EGFR, HER2, HER4*	Boehringer Ingelheim	40 mg once daily
**Cabozantinib (Cabometyx)**	*MET, VEGFR1-3, AXL, RET, ROS1, TYRO3, MER, KIT, TRKB, FLT-3, TIE2*	Exelixis, Inc	60 mg once daily

Abbreviation: FDA: Food and Drug Administration.

### Statistical analyses

Descriptive statistics, including mean, median, standard deviations, and ranges for all continuous measures were tabulated and reported. Overall response rate (ORR) was defined as the proportion of patients with confirmed complete response or partial response according to the Response Evaluation Criteria in Solid Tumors (RECIST), version 1.1. Clinical benefit rate (CBR) was defined as the proportion of patients with complete response, partial response, or stable disease ≥6 months according to RECIST v1.1. For ORR and CBR, patients without a post-baseline tumor assessment were classified as not evaluable. Time to treatment failure (TTF) was measured from the first day of treatment until the patient was removed from the study for TRAEs, progressive disease, patient/investigator choice, or death. Progression-free survival was defined as the time from the first day of study drug administration (Day 1) to progressive disease as defined by the RECIST v1.1 or death on study. Patients who were alive and free from progressive disease were censored at the date of the last tumor assessment. For ORR and CBR, the estimates and the associated 95% confidence intervals (CI) (based on the Clopper-Pearson method) in each treatment group were calculated. For PFS, Kaplan-Meier curves were generated and the median time to event and the associated 95% CIs were provided. Safety was assessed through the analysis of the reported incidence of TRAEs, and those with onset on or after the initiation of therapy were graded according to the National Cancer Institute Common Terminology Criteria for Adverse Events Version 4.03.

## Results

### Patient and disease characteristics

This study enrolled 100 patients across 4 tumor arms: NSCLC (*n* = 39), upper aerodigestive tract cancers (*n* = 9), non-colon gastrointestinal cancers (*n* = 42), and urothelial carcinoma (*n* = 10). The median age of patients was 71 years; 58.0% were male; 93.0% were White, 5.0% were Black or African American, and 2.0% were other races; 92.0% were not Hispanic or Latino (Table 2). Seventy percent of patients had an ECOG performance status of 1. All patients had metastatic disease at study entry, with a median of 3 metastatic sites and a maximum of 5 sites. Across tumor types, the most prevalent genomic alterations were *HER2* (28%), *KRAS* (22%), *BRAF* (14%), and *FGFR1* (10%). Additional alterations found in smaller subsets included *EGFR*, *FGFR1*, *MET*, and *KIT*. The median number of previous treatment regimens in the metastatic setting was 3, with some patients receiving up to 8 prior regimens.

**Table 2.** Demographics summary.

Demographic characteristic	NSCLC (*N* = 39)	Upper aerodigestive tract (*N* = 9)	Non-colon GI (*N* = 42)	Urothelial (*N* = 10)	Total (*N* = 100)
**Age (years)**					
** Mean (SD)**	69.5 (7.7)	66.1 (11.99)	68.5 (10.14)	75.3 (8.38)	69.3 (9.39)
** Median, (min, max)**	70 (53, 87)	64 (48, 83)	70.5 (43, 84)	78 (62, 87)	71 (43, 87)
**Sex, *n* (%)**					
** Male**	14 (35.9)	8 (88.9)	29 (69.0)	7 (70.0)	58 (58.0)
** Female**	25 (64.1)	1 (11.1)	13 (31.0)	3 (30.0)	42 (42.0)
**Ethnicity, *n* (%)**					
** Hispanic or Latino**	1 (2.6)	0	4 (9.5)	0	5 (5.0)
** Not Hispanic or Latino**	36 (92.3)	9 (100)	37 (88.1)	10 (100)	92 (92.0)
** Unknown**	2 (5.1)	0	1 (2.4)	0	3 (3.0)
**Race, *n* (%)**					
** Black or African American**	2 (5.1)	0	3 (7.1)	0	5 (5.0)
** White**	35 (89.7)	9 (100)	39 (92.9)	10 (100)	93 (93.0)
** Other**	2 (5.1)	0	0	0	2 (2.0)

Abbreviations: GI, gastrointestinal; max, maximum; min, minimum; *N*, number of patients; NSCLC, non-small cell lung cancer; SD, standard deviation.

### Treatment received

Fifty-four patients received regorafenib, 35 patients received afatinib, and 11 patients received cabozantinib. Patients were on study treatment for a median of 2.8 (range: 0.5, 44) months. The main reasons for discontinuation were progressive disease (70%; regorafenib = 63%, afatinib = 77%, cabozantinib = 73%) and TRAEs (20%; regorafenib = 26%, afatinib = 14%, cabozantinib = 9%). The median treatment duration was 12 weeks for regorafenib, 10.7 weeks for afatinib, and 24.1 weeks for cabozantinib. Some patients experienced long treatment durations, with 1 patient treated with regorafenib receiving treatment for 193 weeks, 1 patient treated with afatinib receiving treatment for 60 weeks, and 1 patient treated with cabozantinib receiving treatment for 94 weeks. A high percentage of patients on each medication required dose interruptions due to TRAEs (regorafenib = 65%, afatinib = 69%, cabozantinib = 82%). Additionally, rates of dose reductions due to TRAEs for each medication were 43% for regorafenib, 51% for afatinib, and 36% for cabozantinib.

### Efficacy

The overall best response rates are shown in Table 3. In the intent-to-treat population, the ORR for the NSCLC arm was 10.3% (2.9, 24.2 [95% CI]) and the CBR was 15.4% (5.9, 30.5 [95% CI]). For the non-colon gastrointestinal cancers arm, the ORR was 7.1% (1.5, 19.5 [95% CI]), and the CBR was 21.4% (10.3, 36.8 [95% CI]). The upper aerodigestive tract cancers and urothelial carcinoma arms had 1 unconfirmed partial response each, but no confirmed responses.

**Table 3.** Summary of best overall response and related rates of tumor response.

Response	NSCLC (*N* = 39)	Upper aerodigestive tract (*N* = 9)	Non-colon GI (*N* = 42)	Urothelial (*N* = 10)	Total (*N* = 100)
**Complete response, *n* (%)**	0	0	1 (2.4)	0	1 (1.0)
**Partial response, *n* (%)**	4 (10.3)	1 (11.1)	2 (4.8)	1 (10.0)	8 (8.0)
**Stable disease, *n* (%)**	13 (33.3)	2 (22.2)	16 (38.1)	3 (30.0)	34 (34.0)
**Progressive disease, *n* (%)**	10 (25.6)	5 (55.6)	17 (40.5)	4 (40.0)	36 (36.0)
**Not evaluable, *n* (%)**	2 (5.1)	0	0	0	2 (2.0)
**Missing, *n* (%)**	10 (25.6)	2 (22.2)	6 (14.3)	1 (10.0)	19 (19.0)
**Objective response rate, *n* (%)**	4 (10.3)	0	3 (7.1)	0	7 (7.0)
** 95% confidence interval**	(2.9, 24.2)	(0.0, 30.8)	(1.5, 19.5)	(0.0, 33.6)	(2.9, 13.9)
**Clinical benefit rate (CR + PR + SD >180 days), *n* (%)**	6 (15.4)	0	9 (21.4)	1 (10.0)	16 (16.0)
** 95% confidence interval**	(5.9, 30.5)	(0.0, 30.8)	(10.3, 36.8)	(0.3, 48.2)	(9.4, 24.7)

Abbreviations: CR, Complete Response; GI, gastrointestinal; N, number of patients; NSCLC, non-small cell lung cancer; PR, partial response; SD, stable disease.

Eighty-one patients had a post-baseline disease assessment. Among patients with NSCLC, 4 (13.8%) had a partial response, 13 (44.8%) had stable disease, 10 (34.5%) had progressive disease, and 2 (6.9%) were not evaluable. Among patients with upper aerodigestive tract cancers, 1 (12.5%) had an unconfirmed partial response, 2 (25%) had stable disease, and 5 (62.5%) had progressive disease. In the non-colon gastrointestinal cancers arm, 1 patient (2.8%) had a complete response, 2 patients (5.6%) had a partial response, 16 patients (44.4%) had stable disease, and 17 patients (47.2%) had progressive disease. Among patients with urothelial carcinoma, 1 (12.5%) had an unconfirmed partial response, 3 (37.5%) had stable disease, and 4 (50%) had progressive disease (Table S1).

Across treatment arms, patients were observed for genetic mutations. In the regorafenib group, 22 patients (40.7%) had a *KRAS* mutation, and 14 patients (25.9%) had a *BRAF* mutation. In the afatinib group, 28 patients (80.0%) had a *HER2* mutation, and 7 patients (20.0%) had an *EGFR* mutation, while in the cabozantinib group, 3 patients (27.3%) had a *MET* mutation, and 2 patients (18.2%) each had an *AXL*, a *KIT*, and a *RET* mutation, respectively (Table 4).

**Table 4.** Summary of genomic alterations by drug received.

*Alterations*	Regorafenib (N = 54)	Afatinib (N = 35)	Cabozantinib (N = 11)	Total (N = 100)
***Abl***	1 (1.9)	0	0	1 (1.0)
***AXL***	0	0	2 (18.2)	2 (2.0)
			1 (9.1)	1 (1.0)
***BRAF***	14 (25.9)	0	0	14 (14.0)
	3 (5.6)			3 (3.0)
***EGFR***	0	7 (20.0)	0	7 (7.0)
***FGFR1***	9 (16.7)	1 (2.6)	0	10 (10.0)
***FGFR2***	2 (3.7)	0	0	2 (2.0)
	1 (1.9)			1 (1.0)
***FLT-3***	0	0	1 (9.1)	1 (1.0)
***HER2***	0	28 (80.0)	0	28 (28.0)
		1 (2.9)		(1.0)
***HER4***	0	2 (5.3)	0	2 (2.0)
***HRAS***	2 (3.7)	0	0	2 (2.0
***KIT***	4 (7.4)	0	2 (18.2)	6 (6.0)
***KRAS***	22 (40.7)	0	0	22 (22.0)
***MET***	0	0	3 (27.3)	3 (3.0)
			1 (9.1)	1 (1.0)
***PDGFRα***	2 (3.7)	0	0	2 (2.0)
***PDGFRβ***	1 (1.9)	0	0	1 (1.0)
***RET***	0	0	2 (18.2)	2 (2.0)
***ROS1***	0	0	1 (9.1)	1 (1.0)
***Trk2A***	1 (1.9)	0	0	1 (1.0)
**Total**	58	38	11	107^a^

Patients with a response are noted in bold type.

a *N*  =  107 because patients could have more than 1 alteration.

Among patients having a complete or partial response, 3 patients (5.6%) in the regorafenib group had a *BRAF* mutation, and 1 patient (1.9%) had an *FGFR2* mutation; 1 patient (2.9%) in the afatinib group had a *HER2* mutation, and 1 patient (9.1%) each in the cabozantinib group had an *AXL* mutation and a *MET* mutation, respectively (Table 4).

The median PFS was 2.9 months (2.4, 3.9 [95% CI]); across study arms, the median PFS was 3.2 months (2.7, 4.4 [95% CI]) for NSCLC, 2.8 months (1.8, 8.6 [95% CI]) for upper aerodigestive tract cancers, 3.1 months (2.3, 4.2 [95% CI]) for non-colon gastrointestinal malignancies, and 1.9 months (1.5, 3.3 [95% CI]) for urothelial carcinoma. At 6 months, PFS probabilities were 20.6% (9.1, 35.3 [95% CI]) for NSCLC, 29.6% (5.2, 60.7 [95% CI]) for upper aerodigestive tract cancers, 29.8% (16.6, 44.2 [95% CI]) for non-colon gastrointestinal cancers, and 20.0% (3.1, 47.5 [95% CI]) for urothelial carcinoma. The 12-month PFS rates demonstrated sustained response in 3 arms: 7.4% (1.4, 20 [95% CI]) for NSCLC, 14.8% (0.8, 46.8 [95% CI]) for upper aerodigestive tract cancers, and 8.1% (2.1, 19.5 [95% CI]) for non-colon gastrointestinal malignancies; none of the patients with urothelial carcinoma remained progression-free at this timepoint (Table 5).

**Table 5.** Progression-free survival and time to treatment failure by study arm.

Parameter	NSCLC (*N* = 39)	Upper aerodigestive tract (*N* = 9)	Non-colon GI (*N* = 42)	Urothelial (*N* = 10)	Total (*N* = 100)
**Progression-free survival**					
** Number of patients with PFS event, *n* (%)**	32 (82.1)	8 (88.9)	39 (92.9)	10 (100.0)	89 (89.0)
** Number of patients censored, *n* (%)**	7 (17.9)	1 (11.1)	3 (7.1)	0 (0.0)	11 (11.0)
** Median (95% CI) (months)**	3.2 (2.7, 4.4)	2.8 (1.8, 8.6)	3.1 (2.3, 4.2)	1.9 (1.5, 3.3)	2.9 (2.4, 3.9)
**PFS probability estimate**					
** 6 months (95% CI)**	20.6 (9.1, 35.3)	29.6 (5.2, 60.7)	29.8 (16.6, 44.2)	20.0 (3.1, 47.5)	25.2 (16.8, 34.4)
** 12 months (95% CI)**	7.4 (1.4, 20.1)	14.8 (0.8, 46.8)	8.1 (2.1, 19.5)	N/A	7.4 (3.1, 14.3)
** 18 months (95% CI)**	3.7 (0.3, 15.4)	N/A	2.7 (0.2, 12.1)	N/A	2.5 (0.5, 7.7)
** 24 months (95% CI)**	3.7 (0.3, 15.4)	N/A	N/A	N/A	1.2 (0.1, 6.0)
** 30 months (95% CI)**	N/A	N/A	N/A	N/A	N/A
**Time to treatment failure**					
** Number of patients with TTF event, *n* (%)**	37 (94.9)	9 (100)	41 (97.6)	10 (100)	97 (97.0)
** Number of patients censored, *n* (%)**	2 (5.1)	0	1 (2.4)	0	3 (3.0)
** Median (95% CI) (months)**	2.1 (1.6, 2.8)	2.0 (1.0, 4.3)	1.9 (1.8, 3.5)	1.8 (1.1, 1.9)	1.9 (1.9, 2.5)
**TTF probability estimate**					
** 6 months (95% CI)**	11.3 (3.6, 23.9	11.1 (0.6, 38.8)	22.0 (10.9, 35.6)	10.0 (0.6, 35.8)	15.8 (9.3, 23.8)
** 12 months (95% CI)**	2.8 (0.2, 12.5)	11.1 (0.6, 38.8)	4.9 (0.9, 14.5)	N/A	4.2 (1.4, 9.6)
** 18 months (95% CI)**	N/A	N/A	2.4 (0.2, 11.1)	N/A	1.1 (0.1, 5.1)

Abbreviations: CI, confidence interval; GI, gastrointestinal; N, number of patients; N/A, not applicable; NSCLC, non-small cell lung cancer; PFS, progression-free survival; TTF, time to treatment failure.

The median TTF was 1.9 months (1.9, 2.5 [95% CI]), and 97% of patients had their cancer progress or had to stop treatment due to toxicity. Across the 4 arms, the median TTFs were as follows: 2.1 months (1.6, 2.8 [95% CI]) for NSCLC; 2 months (1.0, 4.3 [95% CI]) for upper aerodigestive tract cancers; 1.9 months (1.8, 3.5 [95% CI]) for non-colon gastrointestinal cancers; and 1.8 months (1.1, 1.9 [95% CI]) for urothelial carcinoma (Table 5). At 6 months, only 15.8% of patients (9.3, 23.8 [95% CI]) were still benefiting from treatment. By 12 and 18 months, just 4.2% (1.4, 9.6 [95% CI]) and 1.1% (0.1, 5.1 [95% CI]) of patients, respectively, remained on treatment without progression or intolerable toxicity (Table 5).

### Toxicity

Among patients taking regorafenib, 90.7% (*n* = 49) experienced some grade of TRAEs; any-grade TRAEs occurring in >20% of patients included fatigue (51.9%), nausea (33.3%), decreased appetite (31.5%), diarrhea (24.1%), and dehydration (22.2%). Among patients taking afatinib, 85.7% (*n* = 30) experienced some grade of TRAEs; any-grade TRAEs occurring in >20% of patients were diarrhea (77.1%), nausea (37.1%), and fatigue (25.7%). All 11 patients (100%) taking cabozantinib experienced some grade of TRAEs. Treatment-related adverse events (any-grade) occurring in >20% of patients included diarrhea, fatigue (45.5%), nausea (45.5% each); stomatitis and weight loss (36.4% each); and palmar-plantar erythrodysesthesia syndrome, alanine aminotransferase increased, and aspartate aminotransferase increased (27.3% each) (Table 6).

**Table 6.** Treatment-related adverse events in ≥10% of patients.

Treatment-related adverse events	Grade 1	Grade 2	Grade 3	Grade 4	Grade 3+	Total, *n* (%)
		
	Number of patients, *n* (%)					
**Regorafenib (*n* = 54)**						
** Any AE**	5 (9.3)	17 (31.5)	25 (46.3)	2 (3.7)	27 (50.0)	49 (90.7)
** Fatigue**	12 (22.2)	18 (33.3)	11 (20.4)	0	11 (20.4)	28 (51.9)
** Nausea**	10 (18.5)	9 (16.7)	1 (1.9)	0	1 (1.9)	18 (33.3)
** Decreased appetite**	9 (16.7)	9 (16.7)	2 (3.7)	0	2 (3.7)	17 (31.5)
** Diarrhea**	10 (18.5)	4 (7.4)	2 (3.7)	0	2 (3.7)	13 (24.1)
** Dehydration**	2 (3.7)	10 (18.5)	1 (1.9)	0	1 (1.9)	12 (22.2)
** Stomatitis**	4 (7.4)	6 (11.1)	0	0	0	9 (16.7)
** Constipation**	4 (7.4)	4 (7.4)	0	0	0	8 (14.8)
** Dysphonia**	6 (11.1)	2 (3.7)	0	0	0	8 (14.8)
** Aspartate amino transferase increased**	5 (9.3)	2 (3.7)	3 (5.6)	0	3 (5.6)	7 (13.0)
** Blood bilirubin increased**	2 (3.7)	5 (9.3)	3 (5.6)	2 (3.7)	3 (5.6)	7 (13.0)
** Hypertension**	0	4 (7.4)	4 (7.4)	0	4 (7.4)	7 (13.0)
** Rash**	3 (5.6)	3 (5.6)	1 (1.9)	0	1 (1.9)	7 (13.0)
** Weight decreased**	5 (9.3)	3 (5.6)	0	0	0	7 (13.0)
** Mucosal inflammation**	5 (9.3)	3 (5.6)	1 (1.9)	0	1 (1.9)	6 (11.1)
**Afatinib (*n* = 35)**						
** Any AE**	4 (11.4)	7 (20.0)	18 (51.4)	1 (2.9)	19 (54.3)	30 (85.7)
** Diarrhea**	21 (60.0)	17 (48.6)	13 (37.1)	0	13 (37.1)	27 (77.1)
** Nausea**	7 (20.0)	9 (25.7)	0	0	0	13 (37.1)
** Fatigue**	5 (14.3)	7 (20.0)	3 (8.6)	0	3 (8.6)	9 (25.7)
** Stomatitis**	2 (5.7)	5 (14.3)	0	0	0	7 (20.0)
** Vomiting**	3 (8.6)	5 (14.3)	0	0	0	7 (20.0)
** Decreased appetite**	2 (5.7)	3 (8.6)	1 (2.9)	0	1 (2.9)	6 (17.1)
** Mucosal inflammation**	5 (14.3)	2 (5.7)	0	0	0	6 (17.1)
** Dehydration**	0	5 (14.3)	2 (5.7)	0	2 (5.7)	5 (14.3)
** Dermatitis acneiform**	5 (14.3)	1 (2.9)	0	0	0	5 (14.3)
** Anemia**	1 (2.9)	3 (8.6)	1 (2.9)	0	1 (2.9)	4 (11.4)
** Rash**	4 (11.4)	3 (8.6)	0	0	0	4 (11.4)
**Cabozantinib (*n* = 11)**						
** Any AE**	1 (9.1)	6 (54.5)	4 (36.4)	0	4 (36.4)	11 (100.0)
** Diarrhea**	4 (36.4)	2 (18.2)	1 (9.1)	0	1 (9.1)	5 (45.5)
** Fatigue**	1 (9.1)	4 (36.4)	0	0	0	5 (45.5)
** Nausea**	2 (18.2)	3 (27.3)	0	0	0	5 (45.5)
** Stomatitis**	1 (9.1)	4 (36.4)	1 (9.1)	0	1 (9.1)	4 (36.4)
** Weight loss**	1 (9.1)	3 (27.3)	0	0	0	4 (36.4)
** Alanine aminotransferase increased**	3 (27.3)	1 (9.1)	0	0	0	3 (27.3)
** Aspartate aminotransferase increased**	2 (18.2)	2 (18.2)	1 (9.1)	0	1 (9.1)	3 (27.3)
** Palmar-plantar erythrodysesthesia syndrome**	2 (18.2)	3 (27.3)	0	0	0	3 (27.3)
** Blood alkaline phosphataseincreased**	2 (18.2)	0	0	0	0	2 (18.2)
** Decreased appetite**	1 (9.1)	1 (9.1)	0	0	0	2 (18.2)
** Dehydration**	0	2 (18.2)	0	0	0	2 (18.2)
** Thrombocytopenia**	2 (18.2)	0	0	0	0	2 (18.2)
** Vomiting**	2 (18.2)	0	0	0	0	2 (18.2)

Abbreviation: AE, adverse event.

Among patients taking regorafenib, 8 patients (14.8%) reported 11 serious TRAEs, which included duodenal hemorrhage, pneumoperitoneum, weakness, asthenia, fever, increased bilirubin levels, nervous system disorder, acute heart failure, acute diverticulitis, dyspnea, and dehydration. All serious TRAEs ranged in severity from Grade 2 to Grade 4. For the serious TRAEs of duodenal hemorrhage and dyspnea, the drug was withdrawn; for the serious TRAEs of dehydration and asthenia, doses were interrupted. Among patients taking afatinib, 3 (8.6%) reported 4 serious TRAEs (all Grade 3), which included dehydration (2), diarrhea (1), and fatigue (1). Among patients taking cabozantinib, only 1 (9.1%) reported a serious TRAE (Grade 2 dizziness), which needed dose interruption.

Thirty-two patients (32%) died during this study; 23 patients (23%) died due to progressive disease, 4 patients (4%) died due to adverse events (AEs), and 5 patients (5%) died due to unknown causes. In the NSCLC arm, 9 patients (23.1%) died due to progressive disease, 2 patients (5.1%) died due to AEs, and 3 patients (7.7%) died due to unknown reasons. In the upper aerodigestive tract cancer arm, 3 patients (33.3%) died due to progressive disease. In the non-colon gastrointestinal cancers arm, 10 patients (23.8%) died due to progressive disease, 2 patients (4.8%) died due to AEs, and 1 patient (2.4%) died due to unknown reasons. In the urothelial carcinoma arm, 1 patient (10.0%) died due to progressive disease, and 1 patient (10.0%) died due to unknown reasons.

## Discussion

In this analysis, we evaluated the preliminary antitumor activity of 3 commercially available molecularly matched targeted therapies, regorafenib, afatinib, and cabozantinib, in patients who had disease progression after first-line treatment for NSCLC, upper aerodigestive tract cancers, non-colon gastrointestinal cancers, or urothelial carcinoma. Across cancer types, we observed an ORR of 7.0%, a CBR of 16.0%, and a median PFS of 2.9 months (2.4, 3.9 [95% CI]). Toxicities were common, with >85% of patients experiencing any adverse event for each of the medications.

The overall ORR of 7.0%, as well as the ORRs for patients with NSCLC (10.3%) and non-colon gastrointestinal cancers (7.1%), broadly align with findings from other studies across various cancer types (range, 7% to 14%).^15–19^ The overall median PFS was 2.9 months and ranged from 1.9 months for urothelial carcinoma to 3.2 months for NSCLC. These findings were also consistent with those of other studies.^12^^,^^15^^,^^17^^,^^20^ The overall CBR of 16.0% was lower than the disease control rates reported in other studies, which have ranged from 24% to 37%.^15–19^ However, this could be attributed to differences in definitions of outcomes across studies. In this study, CBR was defined as the proportion of patients with overall response or stable disease ≥6 months; whereas, analyses from the TAPUR study defined disease control rates as overall response or stable disease for at least 16 weeks duration.^15^^,^^16^^,^^18^^,^^19^ Collectively, these findings indicate modest effects of regorafenib, afatinib, and cabozantinib when used as molecularly matched targeted therapies in patients with NSCLC, upper aerodigestive tract cancers, non-colon gastrointestinal cancers, and urothelial carcinoma in the second-line setting.

Several factors could account for the modest effects observed. These include suboptimal matching, as drugs were matched to genomic alterations simplistically as “present” or “absent” and did not consider the nature and genomic position of the alterations. However, the importance of specific changes even in the same gene differs. For instance, BRAF V600E alterations predict MEK inhibitor benefit more consistently than non-V600E changes.^21^ Subgroup analyses and preclinical data are needed to refine matching logic and differentiate drivers from bystanders. Additionally, treatment responses did not take into account the histology of the tumor or the prior treatments received, which could impact outcomes. This study exclusively focused on patients in the second-line setting, and it is possible that these treatments could have stronger effects when administered in the first-line setting. None of the 3 drugs used in this study are the most target-specific antibodies on the market, which may be a variable in the response. Relatedly, combining targeted agents may be needed to overcome inherent resistance.

Another limitation of this study was its sample size; larger cohorts can enable a more rigorous assessment of differences in efficacy between cancer types. Additionally, 19 patients did not have a post-baseline disease assessment.

The study was also limited by the timing of NGS testing and the absence of data stratifying patients according to NGS timing. Reliance on baseline testing alone may underestimate molecular alterations targetable by agents in the second-line setting; however, mixed NGS timing does not account for differences in genomic profiles between initial diagnosis and relapse caused by treatment-induced resistance and clonal evolution, changes necessitating re-biopsy and re-genotyping at the time of progression.

Despite these limitations, this study provides important real-world data on matching commercially available therapies to genomic alterations for a variety of cancer types. Future studies could test the efficacy of combining targeted therapies or adding immunotherapy. Additionally, comparing the molecular features of patients showing exceptional benefit from treatment, patients with stable disease, and patients with disease progression could further refine predictive relationships.

## Conclusions

Regorafenib, afatinib, and cabozantinib had modest effects when used as targeted therapies matched to genomic alterations in patients with NSCLC, upper aerodigestive tract cancers, non-colon gastrointestinal cancer, and urothelial carcinoma in the second-line setting. Future research examining approaches that more precisely match available therapies with genomic alterations and that combine targeted therapies or include immunotherapy is needed to advance the evidence-base for precision oncology.

## Supplementary Material

oyag279_Supplementary_Data

## Data Availability

The data that support the findings of this study are available upon request from the corresponding author. The data are not publicly available due to privacy restrictions.
