# Supplementary material for: A phase II study to evaluate the efficacy of commercially available molecularly matched targeted therapies in the second-line setting
Source: Oncologist. 2026 Jul 22;31(9):oyag279. doi: 10.1093/oncolo/oyag279 (PMC13600393; doi:10.1093/oncolo/oyag279)
Supplement: Supplementary file 1 [file oyag279_supplementary_data.docx]

**Supplemental Table 1: Summary of Best Overall Response and Related Rates of Tumor Response by Study Arm in Patients with Disease Assessments**

| **Response** | **NSCLC (N=29)** | **Upper Aerodigestive Tract (N=8)** | **Non-colon GI (N=36)** | **Urothelial (N=8)** | **Total (N=81)** |
| --- | --- | --- | --- | --- | --- |
| Complete Response, n (%) | 0 | 0 | 1 (2.8) | 0 | 1 (1.2) |
| Partial Response, n (%) | 4 (13.8) | 0 | 2 (5.6) | 0 | 6 (7.4) |
| Unconfirmed Partial Response,  n (%) | 0 | 1 (12.5) | 0 | 1 (12.5) | 2 (2.5) |
| Stable Disease, n (%) | 13 (44.8) | 2 (25.0) | 16 (44.4) | 3 (37.5) | 34 (42.0) |
| Progressive Disease, n (%) | 10 (34.5) | 5 (62.5) | 17 (47.2) | 4 (50.0) | 36 (44.4) |
| Not Evaluable, n (%) | 2 (6.9) | 0 | 0 | 0 | 2 (2.5) |
|  | | | | | |
| Objective Response Rate, n (%) | 4 (13.8) | 0 | 3 (8.3) | 0 | 7 (8.6) |
| 95% Confidence Interval | (3.9, 31.7) | (0.0, 36.9) | (1.8, 22.5) | (0.0, 36.9) | (3.5, 17.0) |
|  | | | | | |
| Clinical Benefit Rate (CR + PR + SD >180 days), n (%) | 6 (20.7) | 0 | 9 (25.0) | 1 (12.5) | 16 (19.8) |
| 95% Confidence Interval | (8.0, 39.7) | (0.0, 36.9) | (12.1, 42.2) | (0.3, 52.7) | (11.7, 30.1) |

Abbreviations: CR: complete response; GI: gastrointestinal; N: number of patients; NSCLC: non-small cell lung cancer; PR: partial response; SD: Stable Disease.
